# Long-term fertilization and manuring effects on the nexus between sulphur distribution and SOC in an *Inceptisol* over five decades under a finger millet–maize cropping system

**DOI:** 10.1038/s41598-024-60357-3

**Published:** 2024-04-29

**Authors:** B. Gokila, G. Manimaran, D. Jayanthi, K. Sivakumar, G. Sridevi, S. Thenmozhi, M. Elayarajan, A. Renukadevi, R. Sudha, P. Balasubramanian

**Affiliations:** 1 Department of Soil Science & Agrl. Chemistry, Tamil Nadu Agricultural University, Coimbatore, 641 003 India; 2 Department of Agricultural Economics, Tamil Nadu Agricultural University, Coimbatore, 641 003 India; 3 Department of Agronomy, Tamil Nadu Agricultural University, Coimbatore, 641 003 India

**Keywords:** SOC dynamics, FT-IR spectroscopy, Sulphur transformation, Soil enzymes, Plant sciences, Environmental sciences

## Abstract

Our investigation revealed that alterations in sulphur (S) pools are predominantly governed by soil organic carbon (SOC), soil nitrogen (N), microbial biomass, and soil enzyme activities in sandy clay loam (*Vertic Ustropept*) soil. We employed ten sets of nutrient management techniques, ranging from suboptimal (50% RDF) to super-optimal doses (150% RDF), including NPK + Zn, NP, N alone, S-free NPK fertilizers, NPK + FYM, and control treatments, to examine the interrelation of S with SOC characteristics. Fourier-transform infrared (FT-IR) spectroscopy was utilized to analyze the functional groups present in SOC characterization across four treatments: 100% NPK, 150% NPK, NPK + FYM, and absolute control plots. Principal component analysis (PCA) was then applied to assess 29 minimal datasets, aiming to pinpoint specific soil characteristics influencing S transformation. In an *Inceptisol*, the application of fertilizers (100% RDF) in conjunction with 10 t ha^−1^ of FYM resulted in an increase of S pools from the surface to the subsurface stratum (OS > HSS > SO_4_^2−^–S > WSS), along with an increase in soil N and SOC. FT-IR spectroscopy identified cellulose and thiocyanate functional groups in all four plots, with a pronounced presence of carbohydrate—protein polyphenol, sulfoxide (S=O), and nitrate groups specifically observed in the INM plot. The PCA findings indicated that the primary factors influencing soil quality and crop productivity (r^2^ of 0.69) are SOC, SMBC, SMBN, SMBS, and the enzyme activity of URE, DHA, and AS. According to the study, the combined application of fertilizer and FYM (10 t ha^−1^) together exert a positive impact on sulphur transformation, SOC accumulation, and maize yield in sandy clay loam soil.

## Introduction

In India, maize ranks third among the major cereal crops after wheat and rice, accounting for around 10% of the country's total food grain production. In accordance with the^1^ (agricoop.nic), maize is grown on 10.04 million hectares in India, yielding 33.62 million tons of production with an average productivity of 3349 kg ha^−1^. In Tamil Nadu, 2.81 million tonnes of maize were produced on 0.40 million hectares of area, with an average productivity of 7008 kg ha^−1^. In India, only 35% of the maize is used directly as human food; the remaining 65% is used in industry and as poultry feed^2^.

After the primary minerals found in soil, such as N, P, and K, sulphur (S) is the fourth essential component for crop productivity. The synthesis of proteins, lipids, vitamins, and taste compound (methanethiol—odour of garlic, methional—boiled potato, and methanethiol—cooked cabbage) in plants depend on sulphur^3^. Among the amino acids that are utilized to make proteins are methionine (21% S), cysteine (26% S), and cystine (27% S). Because of its function and significance in enhancing crop productivity^4^, S nutrition currently gets more attention in Indian agriculture.

Continuous fertilization and intensive farming can eventually deplete the sulphur (S) reserves in soil over time^5^. An average of 11.4, 29.4, and 17.8% of soils in India were acutely deficient, deficient, and latently deficient in available S, respectively^6^. The most limiting important nutrient in the soil determines the potential production of the crops, and it is crucial to give the plants enough nutrients to reach that potential yield.

In soil, sulfide (S^2−^), elemental sulphur (S^0^), thiosulfate (S_2_O_3_^2−^), tetrathionate (S_4_O_6_^2−^), sulfite (SO_3_^2−^), and sulphate (SO_4_^2−^) are among the inorganic species that may have existed with oxidation states of − 2 through to + 6 in the order of most reduced to most oxidized^7^. The biological oxidation of hydrogen sulfide (H_2_S) to sulphate (SO_4_^2−^) is the primary S transformation in the biogeochemical sulphur cycle. Prokaryotes oxidize reduced inorganic sulfur primarily, and the main byproduct of this oxidation is SO_4_^2−^. Microbes are primarily responsible for the mineralization, immobilization, oxidation, and reduction processes that occur in soil when sulfate is transformed into other forms^8^.

Sulphate (SO_4_^2−^) is the most prevalent form of sulfur that plants and soil microorganisms might absorb, although organic sulphur (S that is directly linked to carbon and ester sulfur) pools are also significant sources of sulphur for plants^9^. According to^10^, soil S is necessary for maintenance of soil fertility, soil organic matter (SOM) stocks management, and soil S is significantly associated with SOC and soil N^11^. Due to S deficiency, soil organic matter (SOM) degradation in semi-arid tropical regions, which is a key factor in the significant degradation of soil quality^12^ also SOM is an complex molecule made up of labile and non-labile pools. Furthermore, Gressel et al.^13^ also claim that the functional groups in the soil organic carbon (SOC) have a big influence on sorption characteristics like cation exchange capacity, which is then reflected in soil fertility. FT-IR spectroscopy has been employed for the examination of the organic carbon's functional groups in soil.

In soil, biochemical properties such as microbial biomass carbon (MBC) and microbial biomass nitrogen (MBN) have a significant correlation with nutrient management practices^14^. Microbes in soil play a significant role in soil nutrient fluxes. The combination of inorganic fertilizers and organic manure has been shown to increase both soil microbial biomass nitrogen (SMBN) and soil microbial biomass carbon (SMBC) in soil, as demonstrated by Juan et al.^15^. Additionally, hydrolytic or oxidative enzymes can catalyze a variety of activities, including the breakdown of organic residues, the cycling of nutrients, the synthesis of organic matter, and the construction of soil structure^16^. And, Acosta-Martinez and Ali Tabatabai^17^ reported that enzymes are involved in the mineralization of P and S and that this process is controlled when there is insufficient soluble P and S.

In this context, the objective of the current study is to determine how intensive cropping system, long-term fertilization, affect the S dynamics, SOC, soil N, microbial biomass (MB) and enzyme activities on *Inceptisol*. In addition, we proposed (a). Long-term fertilization may affect the downward distribution of S and associated pools (b). Long-term fertilization may have an effect on the dynamics of S, which is correlated with SOC and (c). Long-term fertilization may change the microbial biomass and the enzyme activities that affect the dynamics of S.

## Materials and methods

### Site description

As part of a long-term fertilizer experiment that was initiated by the ICAR-All India Coordinated Research Project (AICRP) in 1972 and managed by the Department of Soil Science and Agricultural Chemistry at Tamil Nadu Agricultural University, Coimbatore. The 113th maize crop (2022–2023) in the Finger Millet–Maize Cropping Sequence was grown at Field No. 36 F, Eastern Block Farm, where the current study was conducted. The study site is 426.7 m above mean sea level and is situated at latitude 11° North and longitude 77° East.

### Treatment details

The experimental soil is a calcareous mixed black soil with a sandy clay loam texture. It belongs to the *Inceptisol* (*Vertic Ustropept*) under Periyanaickenpalayam soil series. Ten treatment sets, including 50%NPK, 100%NPK, 150% NPK, 100% NPK + Hand Weeding (HW), 100% NPK + Zn, 100% NP, 100% N, 100% NPK + FYM, 100% NPK (–S free), and absolute control, are employed in this investigation. Each treatment set has four replications for greater accuracy. The normal growing seasons for the finger millet and maize crops in this cropping sequence were June to September (Kharif monsoon season) and January to May (summer), respectively. The method of applying fertilizer are in accordance with the schedule showed in Table 1.

**Table 1:** Schedule and mode of fertilizer application.

Treatments	Finger millet	Maize
	N (urea): P (SSP and DAP): K (MOP) (kg ha^−1^) FYM contains (0.47:0.2:0.5% NPK)	
50% NPK	45:22.5:8.75	67.5:31.25:25
100% NPK	90:45:17.5	135:62.5:50
150% NPK	135:67.5:26.25	202.5:93.75:75
100% NPK + HW	90:45:17.5	135:62.5:50
100% NPK + Zn	90:45:17.5	135:62.5:50 + Zn @25 kg ha^−1^
100% NP	90:45	135:62.5
100% N	90	135
100% NPK + FYM	90:45:17.5 (F) 47:20:50 (FYM)	135:62.5:50 (F) 47:20:50 (FYM)
100% NPK (DAP as a P source)	90:45:17.5	135:62.5:50
Absolute control	–	–

### Soil sample collection

After the maize harvest, soil samples were collected from three distinct depths between the surface and the subsurface, ranging from 0 to 0.15 m, 0.15 to 0.30 m, and 0.30 to 0.45 m. The materials were then processed using a 2 mm sieve after being dried. Standard techniques were used to extract each S species sequentially in order to analyse the S dynamics.

### Plant sample processing

Five plants were randomly sampled from each plot, uprooted, and thoroughly washed to remove any adhering dirt and soil particles. Following this, the samples were air-dried initially and then subjected to further drying in a hot air oven at 60 °C until they reached a constant weight. After oven-drying, the samples were powdered using a Wiley mill and utilized for analysing sulphur uptake in maize. To determine the plant S concentration in maize seed and straw, digestion was performed using a di-acid mixture (HNO_3_:HClO_4_) in 3:2 ratio. Subsequently, the digested samples underwent filtration through Whatman No. 42 filter paper following the method described by Jackson^18^.

### Fractionation of soil sulphur

The method taken to estimate the amount of available sulphur (SO_4_^2−^–S) as described by Williams and Steinbergs^19^. The water soluble sulphur (WSS) by^20^ and Heat Soluble Sulphur (HSS) by Williams and Steinbergs^19^. Total sulphur (TS) was calculated using the acid digestion method, according to Tabatabai^21^, and Organic Sulphur (OS) was estimated as stated by Bardsley and Lancaster^22^.

### Biochemical analysis

The enzyme dehydrogenase (DHA) activities were estimated by measuring tri-phenyl formazan (TPF) in soil^23^ and the spectral intensity was measured at 485 nm. Aryl sulphatase activity (AS) of the soil has measured in the activity of ρ-nitrophenol (ρNP) formed by using potassium ρ-nitrophenylsulphate in soil^24^ and the intensity was read at 400 nm in UV/VIS Spectrophotometer. The extracellular urease activity was assayed the quantity of NH_4_^+^ released during urea hydrolysis by Tabatabai and Bremner^25^ in soil.

The oxidisable portion of SOC has been oxidized by K_2_Cr_2_O_7_ chromic acid wet digestion method attributed as Walkley and Black^26^ method, and the chloroform fumigation method was adopted to analyze the soil microbial biomass carbon (SMBC) and soil microbial biomass nitrogen (SMBN) ascribed by Vance et al.^27^ and Jenkinson^28^ in soil.

### Characterization of SOC functional groups by FT-IR spectroscopy

To characterize soil SOC, four representative samples were collected from different plots: 100% NPK, 150% NPK, 100% NPK + FYM, and control plots. Fourier-transform infrared (FT-IR) spectroscopy (JASCO 6800 Japan), was employed for functional group identification. The infrared and mid-infrared regions (5000–400 cm^−1^ or 2–25 µm) were utilized, with a silicon carbide element (1200 K) serving as the blackbody output. A tungsten-halogen lamp was used as the source for the near-IR (1–2.5 µm or 10,000–4000 cm^−1^), with a quartz envelope for longer wavelengths (5 µm or 2000 cm^−1^); a mercury discharge lamp was used for the far IR, providing higher output than a thermal source. The soil samples were air-dried and sieved through a 2 mm sieve. For the functional group analysis, 300 mg of soil was taken, combined with 900 mg of FT-IR-grade KBr (99%), and ground in an agate mortar. The homogeneous mixture was then transferred into a diffuse reflectance cup without applying pressure, and it was leveled with a microscope glass slide. The FTIR spectra were then measured on an FTIR spectrometer. The FTIR spectroscopy data was graphed using Origin Pro 8.5 software. Subsequently, the raw data underwent baseline correction before being plotted to generate the final graph.

### Soil Quality Index

The methodology used by Andrews et al.^29^ to generate the soil quality index consists of the three steps listed below.

#### Selection of minimum data set

Principal Component Analysis was applied to identify the representative minimum data sets in order to prevent dimensionality^30^. By using a linear combination of indicators to account for the greatest variation within a set, PCA displays distinct principal components, and subsequent PCs are similarly represented. PCs with > 1eigen values were often chosen for MDS, and for each component, 10% of the highly weighed variables are kept in a single PC. When many indicators were kept in a single PC, correlations were used to look at the indicators to see if the variables might be neglected from each PC if they were reductant MDS^29^.

#### Variable transformation and normalization

Each indicator's different dimensions were converted into unitless scores, which ranged from 0 to 1, to represent their respective contributions to soil function and standardize the data. These scores were assigned using standard scoring systems, such as linear and nonlinear scoring functions. The degree to which a greater value is regarded as better or worse depends on how sensitive each indicator is. The assigned score for indicators where a higher value is desirable was set to 1 by dividing the observed value by the highest observed value (denominator). On the other hand, in cases where a lower value is preferred for an indicator, a score of one was determined by dividing the lowest observed value (numerator) by the corresponding observation^31^.

#### Computation of soil quality index

Upon variable normalization, distinct soil quality indices were assigned by means of additive SQI (SQI_A_—^29^) and weighted additive SQI (SQI_W_—^30^);

1 $$\mathrm{SQIw }=\sum_{i=1}^{n}(Wi \times Qi)$$

2 $${\text{SQIA}}=\sum_{i}^{1}\frac{Ni}{n}$$

Where, SQI (Soil Quality Index); W (Assigned Weight of indicators); Q (Score of the indicators).

### Data analysis

To evaluate the variations between treatments and replications, statistical analysis was performed on the experimental data. The significance of variances among treatments was further investigated using Tukey's HSD test, with means compared at a significance threshold of P < 0.05, after one-way ANOVA was used to analyze these differences. After data normalization, PCA was performed using SPSS version 25 to assess the effect of long-term manuring and fertilizer on soil quality. Using a significance level of P < 0.01 and Pearson correlation coefficients, correlations between the experiment's variables were investigated.

### Ethics approval and consent to participate

Our experiment adheres to applicable institutional, national, and international guidelines and legislation

## Results

### Long term fertilisation and intensive cropping on distribution of soil sulphur

#### Sulphate–S

**Figure 1:**
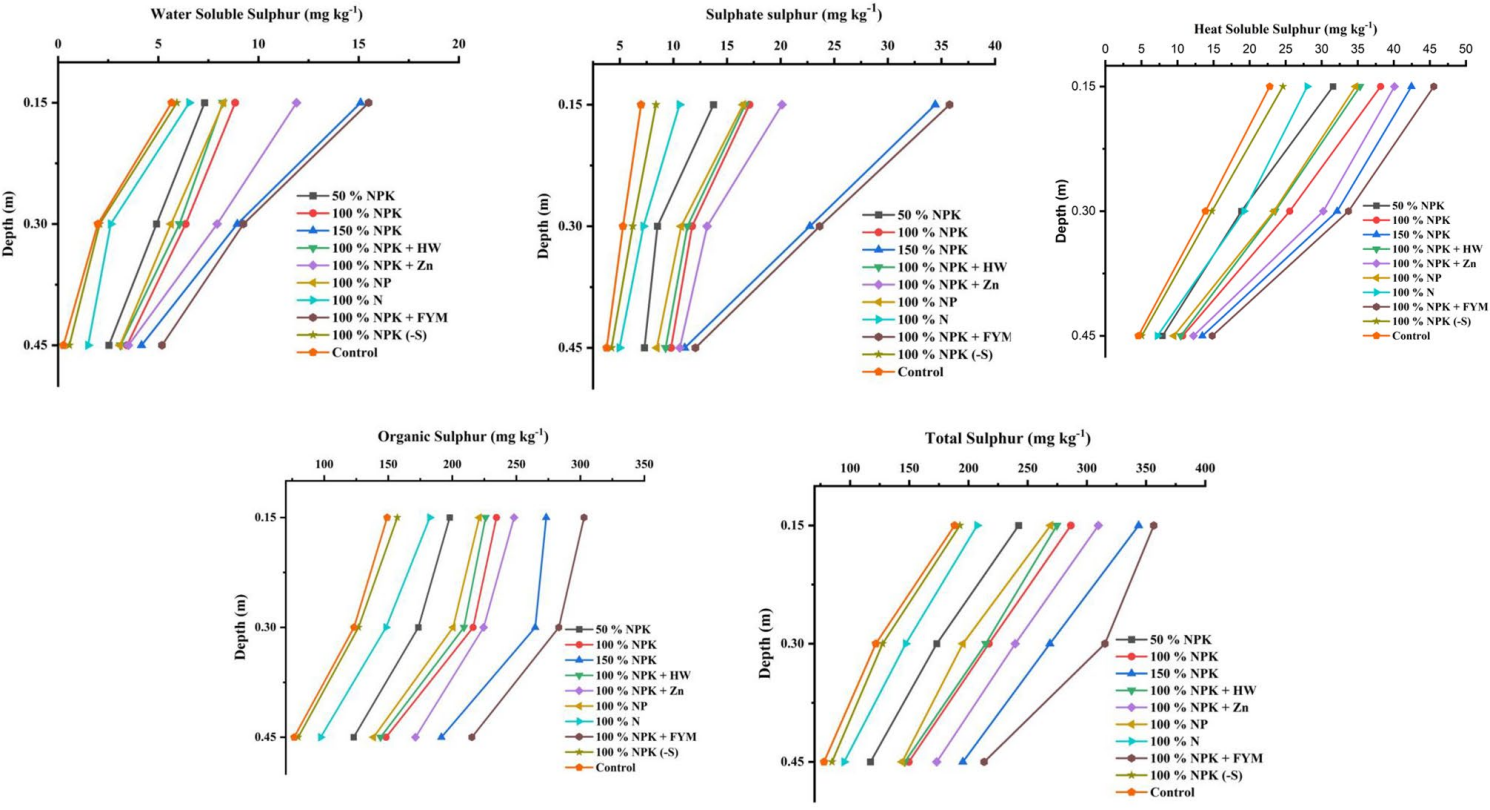
Effect of long term fertilization and manuring on distribution of sulphur fraction with varying depths in sandy clay loam soil. *Sulphur fractions; sulphate sulphur, water soluble sulphur, heat soluble sulphur, organic sulphur and total sulphur.

Sulphate–S (SO_4_^2−^–S) is a form of plant accessible S that is found in soil and the availability has declined from surface to sub surface layers. From surface to sub surface strata, it accounts for 6.82% (0–0.15 m), 6.14% (0.15–0.3 m), and 5.76% (0.3–0.45 m) of the TS respectively (Fig. 1 and Supplementary Table 1). The recommended fertiliser dose increase from 50% NPK to 150% NPK resulted in an increase in soil SO_4_^2−^–S levels of 13.7 to 34.4 mg kg^−1^ (0–0.15 m), 8.5 to 22.7 mg kg^−1^ (0.15–0.3 m), and 7.3 to 11.1 mg kg^−1^ (0.3–0.45 m). Irrespective of the treatments, 100% NPK plus FYM 10 t ha^−1^ (INM) recorded the highest SO_4_^2−^–S content in three depths, measuring 35.8 (0–0.15 m), 23.6 (0.15–0.3 m) and 12.1 mg kg^−1^ (0.3–0.45 m) of S in soil respectively followed by 150% NPK. In sandy clay loam soil, depletion of SO_4_^2−^–S was seen in S free 100% NPK (8.4, 6.2, and 4.2 mg kg^−1^) and 100% N alone (10.6, 7.2, and 5.0 mg kg^−1^) plots as well as control plots (7.0, 5.3, and 73.8 mg kg^−1^).

#### Water soluble sulphur

Water Soluble Sulphur (WSS) represents 3.5, 2.9, and 1.9 percent of the total sulphur in the adjacent layers, respectively. It is a portion of the accessible sulphur fraction that has an easily mineralisable portion in soil (Fig. 1 and Supplementary Table 2). The WSS content decreased from surface to subsurface strata, ranging from 7.37 to 15.1 (0–0.15 m), 4.91 to 8.94 (0.15–0.3 m), and 2.53 to 4.17 mg kg^−1^ (0.3–0.45 m). Continuous application of fertiliser along with FYM 10 t ha^−1^ has resulted in a substantial increase in the WSS content of 15.5, 9.25 and 5.18 mg kg^−1^ followed by super optimal doses (15.1, 8.94 and 4.17 mg kg^−1^) in each respective layers of an *Inceptisol*. The less WSS accumulation was noted in N alone, S free 100% NPK and control plots and other Optimal Doses Are Statistically On Par With Each Other’s.

#### Heat-soluble sulphur

Out of the total sulphur in each stratum, the HSS accounted for 12.9%, 11.7%, and 7.01%, respectively. The HSS content in each of the three layers might rise from 31.6 to 42.4 mg kg^−1^ (0–0.15 m), 18.9 to 32.2 mg kg^−1^ (0.15–0.3 m), and 7.9 to 13.5 mg kg^−1^ (0.3–0.45 m) as a result of applying fertiliser doses from suboptimal to super optimal levels (Fig. 1 and Supplementary Table 3). Continuous addition of NPK plus FYM 10 t ha^−1^ could greatly raise the HSS of 45.5, 33.7 and 14.8 mg kg^−1^ from surface to subsurface stratum. A greater HSS of 42.2, 32.2, and 13.5 mg kg^−1^ was obtained by super optimum dosages after INM, followed by an NPK plus Zn plot. The treatments involving NPK + HW and NP exhibited no statistically significant difference between them. Consistently omission of S supplements resulted in a decrease in HSS in plots treated with N alone, NPK (–S), and control plots.

#### Organic sulphur

Organic sulphur, which makes up 82–83% of the total amount of sulphur in soil, is the most prevalent form (Fig. 1 and Supplementary Table 4). The OS concentration decreased from the surface to the subsurface layers while the fertiliser dose rose from suboptimal (198, 173 and 123 mg kg^−1^) to super optimal level (273, 265 and 192 mg kg^−1^). When comparing the different methods of nutrient management, it was noted that using mineral fertilizer coupled with organic amendments (FYM @ 10 t ha^−1^) led to greater levels of OS in the soil, with values of 303 mg kg^−1^ (0–0.15 m), 283 mg kg^−1^ (0.15–0.3 m), and 215 mg kg^−1^ (0.3–0.45 m). N alone, S free NPK plots, and control plots with sandy clay loam soil were shown to have the least built up of OS content.

#### Total sulphur

Total sulphur, which includes both organic and inorganic forms of sulphur, ranged from 188 mg kg^−1^ in the control to 357 mg kg^−1^ in the 100% NPK + FYM @ 10 t ha^−1^ plots (Fig. 1 and Supplementary Table 5). The total sulphur levels significantly increased in 100% NPK + FYM @ 10 t ha^−1^ plot, 100% NPK + Zn plot, and additional inputs supplied (150% NPK) more than recommended dose. In absolute control plot, the TS concentration decreased from the surface to the subsurface layers, with the lowest values being 188 (0–0.15 m), 122 (0.15–0.3 m), and 78 mg kg^−1^ (0.3–0.45 m) in soil.

### Long term fertilization intensive cropping on SOC and soil microbial biomass (C, N and S) in sandy clay loam soil

#### Soil organic carbon

**Figure 2:**
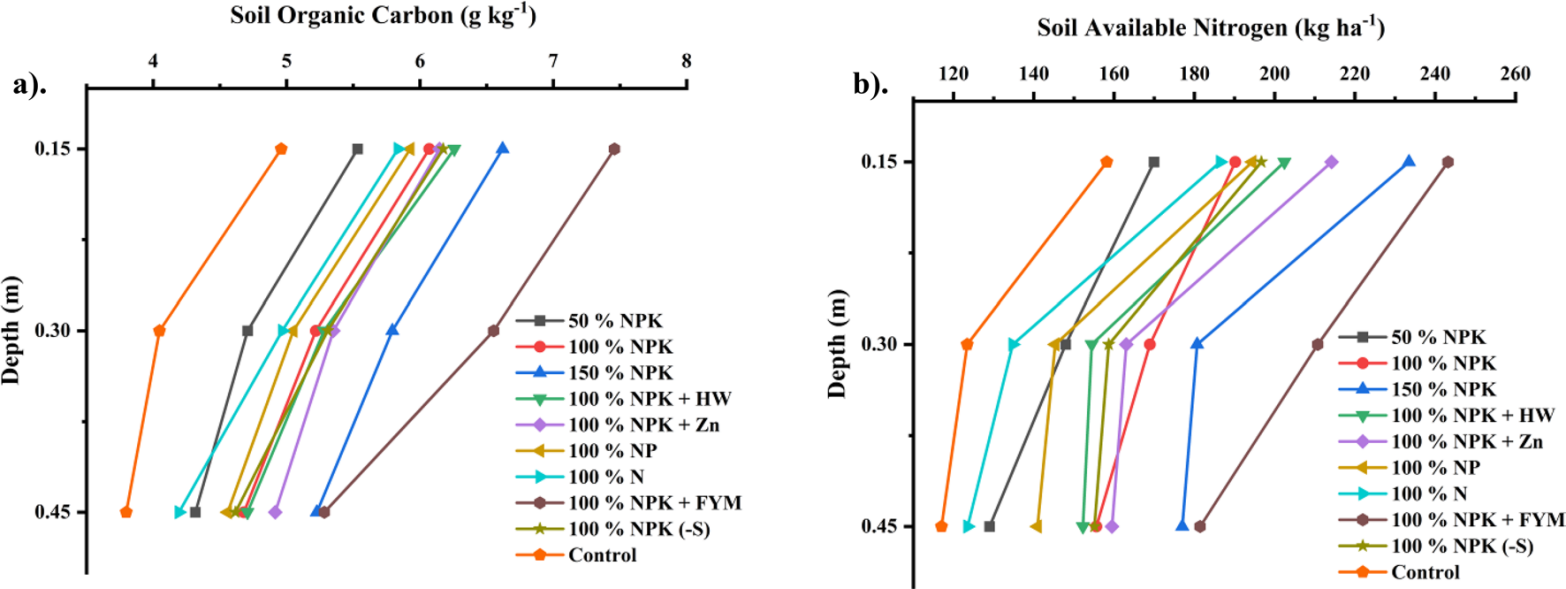
Effect of long term fertilization and manuring on distribution of (**a**) SOC and (**b**) KMnO_4_—N (available) with varying depths in sandy clay loam soil.

SOC is a significant soil property that is closely linked to the biological utilization of nitrogen and sulphur in soil. Fertilizer combined with manure (FYM 10 t ha^−1^) applications resulted in an increase of SOC from 3.0 g kg^−1^ (1972) to 7.46 g kg^−1^ (2023). The decline in SOC is observed from surface to subsurface strata. However, with an increase in fertilizer doses from 50% NPK to 150% NPK, there was an improvement in SOC levels as indicated by the following values: 5.53 to 6.62 (0–0.15 m), 4.71 to 5.79 (0.15–0.3 m), and 4.32 to 5.23 (0.3–0.45 m). The greater SOC of 7.46 g kg^−1^, 6.55 g kg^−1^, and 5.28 g kg^−1^ in each of the three layers (0 to 0.45 m) was recorded in the NPK + FYM plot, followed by super optimum dosages (Fig. 2 and Supplementary Table 6). The other complementary practices like NPK + Zn, NPK + HW, NPK (–S) and NP alone are statistically on par with others. Less SOC accumulation was noticed in all the layers of N alone (5.53, 4.71 and 4.32 g kg^−1^) and control plots (4.96, 4.05 and 3.80 g kg^−1^) respectively.

#### Mineralizable nitrogen

Mineralizable Nitrogen, a crucial primary nutrient, plays a key role in regulating plant growth and metabolism in soil through various nutrient management practices. INM demonstrated notably higher mineralizable nitrogen levels at depths of 0–0.15 m (243 kg ha^−1^), 0.15–0.3 m (211 kg ha^−1^), and 0.3–0.45 m (181 kg ha^−1^). As fertilizer doses increased from suboptimal to super optimal, a substantial increase in N content was observed in sandy clay loam soil (Fig. 2 and Supplementary Table 7). Notably, mineralizable nitrogen levels were lower in plots treated with N alone and control plots, despite five decades of intensive farming in *Inceptisol*.

#### Soil microbial biomass (carbon, nitrogen and sulphur)

In agricultural soil, the amount of soil microbial biomass ranges from 2 to 3% of the total SOM, and the addition of fertilizer or manure can have a significant effect on the SMB of C, N, and S. The increase in SMB of C, N, and S was noticed with 259 to 290 mg kg^−1^, 27.7 to 45.2 mg kg^−1^ and 9.1 to 11.9 mg kg^−1^ respectively, in soil when fertilizer doses were increased from suboptimal to super optimum amounts (Table 2). The addition of 100% NPK with FYM of 10 t ha^−1^ considerably increased the SBMC, SMBN and SMBS of 304, 48.3, and 13.2 mg kg^−1^, respectively followed by super optimal dosages of nutrients (T_3_). The other optimal doses (100%NPK + Zn, 100%NPK + HW, 100%NPK (–S) and 100% NP) were comparable to one another. The SMB of C, N, and S in the soil was lower in the continuous addition of N fertilizers (199, 26.2 and 8.1 mg kg^−1^) and control plot (191, 18.5 and 6.4 mg kg^−1^).

**Table 2:** Effect of continuous fertilisation and cropping on soil microbial biomass and enzyme activities in an *Inceptisol.*

Treatments	SMBC	SMBN	SMBS	AS (µg ρNP g^−1^ soil hr^−1^)	DHA (µg TPF g^−1^ soil day^−1^)	URE (µg NH_4_ g^−1^ soil hr^−1^)
	(µg g^−1^)					
50% NPK	259^*d*^ (± 4.7)	27.7^*d*^ (± 0.57)	9.1^*e*^ (± 0.21)	105^*cd*^ (± 4.0)	9.2^*de*^ (± 0.29)	91^*ef*^ (± 4.1)
100% NPK	281^*bc*^ (± 3.8)	39.0^*b*^ (± 0.22)	11.2^*c*^ (± 0.26)	114^*bc*^ (± 4.0)	11.5^*b*^ (± 0.32)	104^*cde*^ (± 4.2)
150% NPK	290^*ab*^ (± 4.0)	45.2^*a*^ (± 0.57)	11.9^*b*^ (± 0.24)	123^*b*^ (± 3.5)	11.8^*b*^ (± 0.40)	122^*b*^ (± 3.3)
100% NPK + HW	275^*bcd*^ (± 3.9)	38.8^*b*^ (± 0.94)	10.5^*d*^ (± 0.21)	120^*bc*^ (± 4.0)	10.9^*bc*^ (± 0.34)	108^*bcd*^ (± 3.8)
100% NPK + Zn	283^*bc*^ (± 2.7)	41.9^*b*^ (± 0.60)	11.4^*c*^ (± 0.25)	125^*b*^ (± 3.6)	11.3^*bc*^ (± 0.41)	113^*bc*^ (± 4.1)
100% NP	268^*cd*^ (± 3.8)	34.3^*c*^ (± 0.75)	10.7^*d*^ (± 0.24)	120^*bc*^ (± 3.2)	10.8^*bc*^ (± 0.32)	96^*def*^ (± 4.3)
100% N	199^*e*^ (± 4.1)	26.2^*d*^ (± 0.83)	8.1^*g*^ (± 0.23)	95^*de*^ (± 3.6)	9.9^*cd*^ (± 0.26)	85f. (± 3.5)
100% NPK + FYM	304^*a*^ (± 3.6)	48.3^*a*^ (± 0.75)	13.2^*a*^ (± 0.24)	157^*a*^ (± 2.8)	14.3^*a*^ (± 0.33)	143^*a*^ (± 3.9)
100% NPK (-S)	278^*bc*^ (± 2.8)	39.9^*b*^ (± 0.46)	8.6^*f*^ (± 0.27)	98^*de*^ (± 4.1)	11.1^*bc*^ (± .37)	111^*bcd*^ (± 4.1)
Control	191^*e*^ (± 4.3)	18.5^*e*^ (± 0.32)	6.4^*h*^ (± 0.23)	86^*e*^ (± 3.6)	7.7^*e*^ (± 0.38)	57^*g*^ (± 3.2)

In each column, values followed by the common letters are not significantly different at P ≤ 0.05 based on the Tukeys HSD and values followed by ± symbol denotes the standard error of means.

### Soil enzyme activities

#### Aryl sulphatase (AS), dehydrogenase (DHA) and urease (URE)

Application of inorganic fertilizers alone or combined with manure has a significant impact on enzyme activities in soil. The increase of enzyme AS, DHA and URE are recorded in NPK conjunction with 10 t of FYM ha^−1^ (157 µg ρNP g^−1^ soil hr^−1^, 14.3 µg TPF g^−1^ soil day^−1^ and 143 µg NH_4_ g^−1^ soil hr^−1^) followed by 150%NPK (Table 2). Application of fertilisers from 50%NPK doses to 150%NPK doses had increased the AS (105 to 123 µg ρNP g^−1^ soil hr^−1^), DHA (9.2 to 11.8 µg TPF g^−1^ soil day^−1^) and URE (91 to 122 µg NH_4_ g^−1^ soil hr^−1^) activities. Continuous addition of N alone lowered the activities of enzyme AS (95 µg ρNP g^−1^ soil hr^−1^), DHA (9.9 µg TPF g^−1^ soil day^−1^) and URE (85 µg NH_4_ g^−1^ soil hr^−1^) followed by absolute control in soil.

#### Long term fertilisation and intensive cropping on grain yield and S uptake of maize

The application of mineral fertilizers or their combination with manures under intensive cropping greatly increased the yield of both grain and straw in maize (Table 3). The yield of maize grain was highest in NPK + FYM 10 t ha^−1^ (6374 kg ha^−1^), followed by super optimal doses (5276 kg ha^−1^) and NPK + Zn (5106 kg ha^−1^) which increased the yield by 20.8% and 24.8% over 150% NPK and 100% NPK plots. The continuous N fertilized plot and control plot had lower grain yields with 2796 kg ha^−1^ and 1982 kg ha^−1^, respectively.

**Table 3:** Effect of continuous fertilisation and cropping on yield (kg ha^−1^) and sulphur uptake (kg ha^−1^) of maize (COHM6).

Treatments	Grain yield	Straw yield	Total S uptake
50% NPK	3162^*e*^ (± 44)	6184^*d*^ (± 39)	7.6^*f*^ (± 0.12)
100% NPK	5018^*bc*^ (± 53)	8096^*bc*^ (± 142)	12.0^*d*^ (± 0.08)
150% NPK	5276^*b*^ (± 101)	8343^*b*^ (± 83)	16.5^*b*^ (± 0.17)
100% NPK + HW	4873^*cd*^ (± 46)	7822^*c*^ (± 38)	12.5^*d*^ (± 0.10)
100% NPK + Zn	5106^*bc*^ (± 88)	8189^*bc*^ (± 78)	14.0^*c*^ (± 0.22)
100% NP	4622^*d*^ (± 54)	8083^*bc*^ (± 146)	11.9^*d*^ (± 0.04)
100% N	2796^*f*^ (± 33)	6452^*d*^ (± 21)	8.1^*f*^ (± 0.15)
100% NPK + FYM	6374^*a*^ (± 50)	9292^*a*^ (± 109)	19.5^*a*^ (± 0.42)
100% NPK (-S)	4883^*cd*^ (± 70)	7843^*c*^ (± 105)	10.7^*e*^ (± 0.20)
Control	1982^*g*^ (± 43)	4184^*e*^ (± 30)	4.3^*g*^ (± 0.07)

In each column, values followed by the common letters are not significantly different at P ≤ 0.05 based on the Tukeys HSD and values followed by ± symbol denotes the standard error of means.

In terms of total S uptake of the maize crop (grain + straw), NPK coupled with FYM 10t ha^−1^ had the highest S uptake of 19.5 kg ha^−1^, followed by the super optimum dose plot of 16.5 kg ha^−1^. Other optimal doses such as NPK + Zn, NPK + HW, NPK (-S), and NP plots did not reveal any appreciable differences. The control plot and long term exclusion of (N alone) exhibited less S uptake than the plot with sulphur supplementation (SSP as P fertilizer).

#### SOC functional groups by FT-IR spectroscopy

Table 4 displays the FT-IR spectra of soil samples from different treatments in a 50-year-old long-term fertilizer experiment. The absorption band in the wavelength range of 3630 to 3616 cm^−1^ indicates the presence of cellulose functional groups, including O–H and N–H stretching, Si–O–H stretching in clays, and C–O stretching, C–H, and CH_2_ stretching. These functional groups are present in all (control, 100%NPK, 150%NPK and NPK + FYM) treatments. Carbohydrate-protein polyphenol groups, characterized of H-bonded O–H and N–H stretching (including aliphatic primary amines), are observed in the range of 3369 to 3362 cm^−1^ in INM and super-optimal doses. Thiocyanate functional groups (S–C–N stretching) and azide (N=N=N stretching), and/or aliphatic C–H stretching, are evident in the 2102 to 2075 cm^−1^ range in (control, 100%NPK, 150%NPK and NPK + FYM). In the fertilized plot alone (100%NPK, 150%NPK and INM), a peak in the range of 1628 to 1608 cm^−1^ is attributed to imine/oxime functional groups (C=N stretching) and/or conjugated ketones (aromatic C=C stretching or carboxylate C–O asymmetric stretching or C=O stretching). Use of fertilizer, either alone or in combination with manure, is associated with the presence of acetyl groups (carbonyl linked to methyl; CH_3_CO), sulfoxide (S=O), and nitrate groups, which are observed in the 1428 to 1414 cm^−1^ range. Phenolic esters of polysaccharide (C–O–C, C–OH stretching) and/or silicates are evident in the 1135 to 1023 cm^−1^ range in treatments involving INM and optimal fertilization (100%NPK) alone. In general, all plots exhibit the presence of other alkanes, including aromatic C–H and C=C (mostly monosubstituted), in the 995 to 699 cm^−1^ range.

**Table 4:** SOC functional groups by FT-IR spectroscopy.

100%NPK + FYM	Control	100%NPK	150%NPK
**3630 cm**^**−1**^; O–H and N–H stretch, Si–O–H stretch in clays and/or C–O stretch, C–H and CH_2_ stretch (cellulose)^98^	**3627 cm**^**−1**^; O–H and N–H stretch, Si–O–H stretch in clays and/or C–O stretch, C–H and CH_2_ stretch (cellulose)^98^	**3621 cm**^**−1**^; O–H and N–H stretch, Si–O–H stretch in clays and/or C–O stretch, C–H and CH_2_ stretch (cellulose)^98^	**3616 cm**^**−1**^; O–H and N–H stretch, Si–O–H stretch in clays and/or C–O stretch, C–H and CH_2_ stretch (cellulose)^98^
**3362 cm**^**−1**^; O–H stretching bond with carboxylic, alcohol group and, water molecules and N–H Stretching (included aliphatic primary amines) O–H stretching groups of carbohydrate-protein polyphenol^99^	**2086 cm**^**−1**^; S–C–N stretching group of thiocyanate and N=N=N stretching of azide and/or Aliphatic C–H stretch^100^	**2102 cm**^**−1**^; Aliphatic C–H stretch^101^	**3369 cm**^**−1**^; O–H stretching bond with carboxylic, alcohol group and, water molecules and N–H Stretching (included aliphatic primary amines) O–H stretching groups of carbohydrate–protein polyphenol^99^
**2084 cm**^**−1**^; S–C–N stretching group of thiocyanate and N=N=N stretching of azide and/or Aliphatic C–H stretch^100^	**1608 cm**^**−1**^; C=N stretching imine /oxime^102^ (or) aromatic C=C stretch and /or carboxylate C–O asymmetric stretch and/or conjugated ketone C=O stretch^101^	**1617 cm**^**−1**^; C=O stretch C=N stretching imine/oxime^102^	**2075 cm**^**−1**^; S–C–N stretching group of thiocyanate and N=N=N stretching of azide and/or Aliphatic C–H stretch^100^
**1623 cm**^**−1**^; aromatic C=C stretch and /or carboxylate C–O asymmetric stretch and/or conjugated ketone^86^	**1014 cm**^**−1**^; ester phenol C–O–C, C–OH stretch, attributed to polysaccharides or polysaccharides like compounds, silicates^103^	**1420 cm**^**−1**^; aliphatic C–H bend, nitrates, acetyl^101^	**1628 cm**^**−1**^; amide C=O stretch (amide 1) organic nitrates^104^
**1428 cm**^**−1**^; Carboxylic acids and carboxylic groups, H–C–H scissoring, Vibrations of carbonate in calcite and minerals of calcite and dolomite groups, C/H bending vibrations of CH_3_ and CH_2_ groups. malonic and benzoic acid, stretching of amide III of primary amides, S=O stretching of sulfate compound and sulfonyl chloride^108^	**796 cm**^**−1**^ **and 799 cm**^**−1**^; aromatic C–H out of plane bend; increasing wavenumber with increasing degree of substitution^105^	**1023 cm**^**−1**^; ester phenol C–O–C, C–OH stretch, attributed to polysaccharides or polysaccharides like compounds, silicates^103^	**1414 cm**^**−1**^; aliphatic C–H bend, nitrates, acetyl^106^
**1135 cm**^**−1**^; ester phenol C–O–C, C–OH stretch, attributed to polysaccharides or polysaccharides like compounds, silicates^103^		**973 cm**^**−1**^; aromatic C–H out of plane bend; increasing wavenumber with increasing degree of substitution^107^	**988 cm**^**−1**^; CH_2_ out of plane bending terminal methylene, C=C bending alkene monosubstituted^107^
**995 cm**^**−1**^; CH_2_ out of plane bending terminal methylene, C=C bending alkene monosubstituted^107^		**770 cm**^**−1**^; aromatic C–H out of plane bend; increasing wavenumber with increasing degree of substitution^105^	**788 cm**^**−1**^; aromatic C–H out of plane bend; increasing wavenumber with increasing degree of substitution^105^
**890 cm**^**−1**^; aromatic C–H out of plane bend; increasing wavenumber with increasing degree of substitution^105^		**679 cm**^**−1**^; aromatic C–H out of plane bend; increasing wavenumber with increasing degree of substitution^105^	**679 cm**^**−1**^; aromatic C–H out of plane bend; increasing wavenumber with increasing degree of substitution^105^
**799 cm**^**−1**^; aromatic C–H out of plane bend; increasing wavenumber with increasing degree of substitution^105^			

#### Developing of soil quality indices by Principal Component Analysis

In soil suphur transformation, determining critical indicators is a complex task aimed at balancing the source and sink of plant-available nutrients in soil. To simplify this process, PCA analysis was performed to establish the MDS (Minimum Data Set) of various soil parameters that exhibited significant differences among treatments within different ecosystems. In this study, 29 variables were utilized for PCA, and the first two principal components (PCs) with Eigen values greater than 1 were identified as having strong explanatory power (Supplementary Table 8).

From the component matrix, high-scoring variables from the MDS, within the upper 10% in each PC, were selected. These selected variables then underwent Pearson correlation analysis to eliminate redundant variables within the PCs. The first PC exhibited a variability of 88.59%, and the highly weighted variables, including WSS (0.15–0.3 m), HSS (0.15–0.3 m), OS (at three depths), TS (at three depths), soil N (up to 30 cm), SMBS, and AS, were chosen for further analysis^29^. Subsequent PCs had the variability with 6.26%, and included positive variables such as SOC (0–0.15 m, 0.15–0.3 m), SMBC, SMBN, DHA, and URE, which were retained in the PC 2 (Supplementary Table 8).

**Figure 3:**
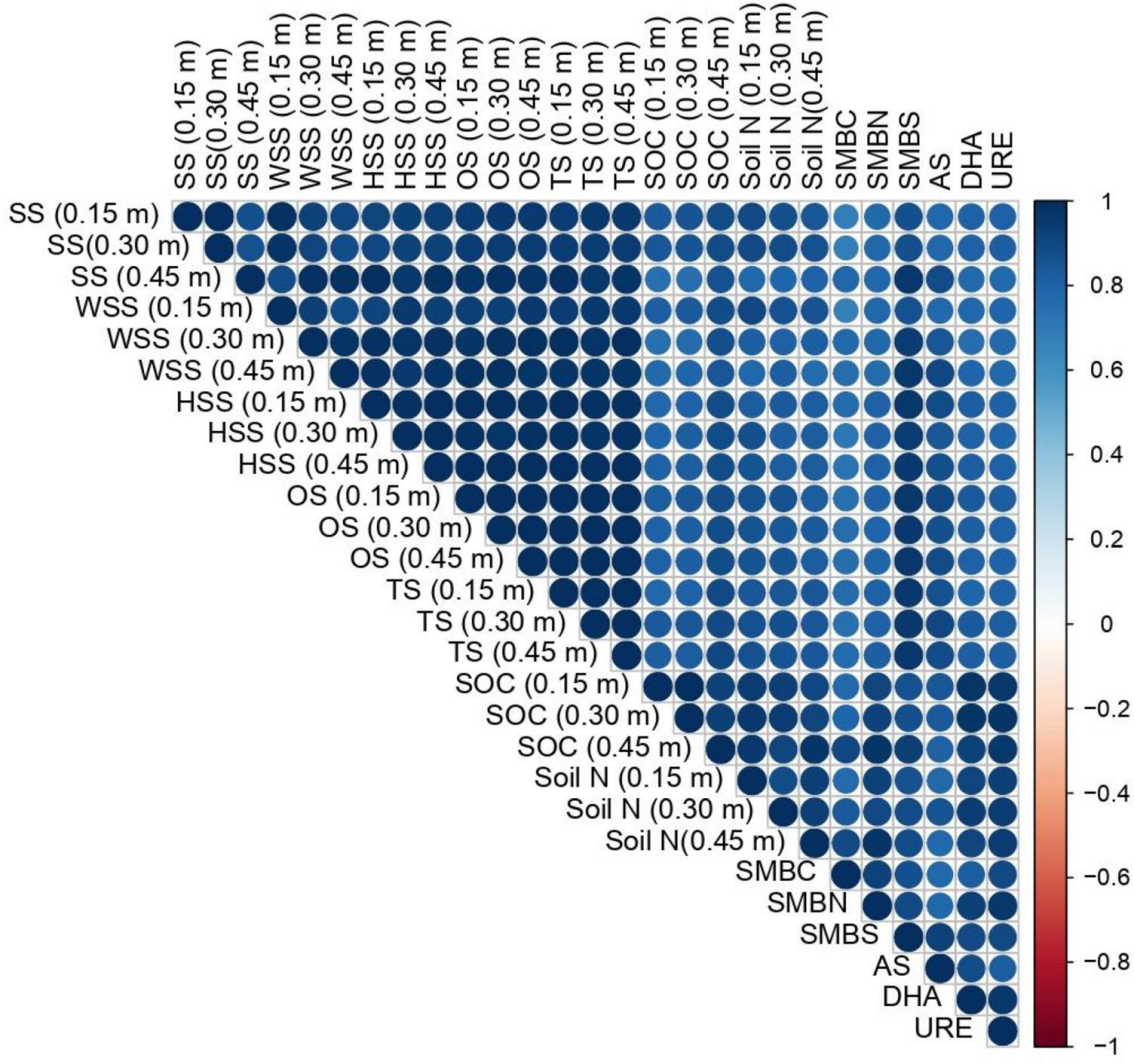
Pearson Correlation Matrix (n) with soil variables in an *Inceptisol* of Finger millet–maize cropping sequence. *SS* sulphate Sulphur, *WSS* water soluble sulphur, *HSS* heat soluble sulphur, *OS* organic sulphur, *TS* total sulphur, *SOC* soil organic carbon, *Soil N* nitrogen, *SMBC* soil microbial biomass carbon, *SMBN* soil microbial biomass nitrogen, *SMBS* soil microbial biomass sulphur, *AS* aryl sulphatase, *DHA* dehydrogenase, *URE* urease. Depths; 0–0.15, 0.15–0.30 and 0.30–0.45 m. (n = 10).

The retained variables were subjected to Pearson correlation analysis to eliminate collinearity, and all variables were then transformed using a linear scoring function. This transformation allowed the variables to be scaled from 0 to 1, and key indicators were categorized as either "Good" or "Bad" based on the criteria outlined in Eqs. 1 and 2 (Supplementary Table 9). Using these transformed variables, different Soil Quality Indices (SQI) were developed, as described in Eqs. 3 and 4, to assess and compare the significance of different treatments. The component matrix also highlighted the importance of certain indicators in relation to sulphur (S) transformation in sandy clay loam calcareous soils. Specifically, S fractions of WSS (0–0.15 m), HSS (0.15–0.3 m), OS (3 depths), and TS (3 depths) were found to be highly correlated with total sulphur uptake and grain yield in *Inceptisol* (Fig. 3).

**Figure 4:**
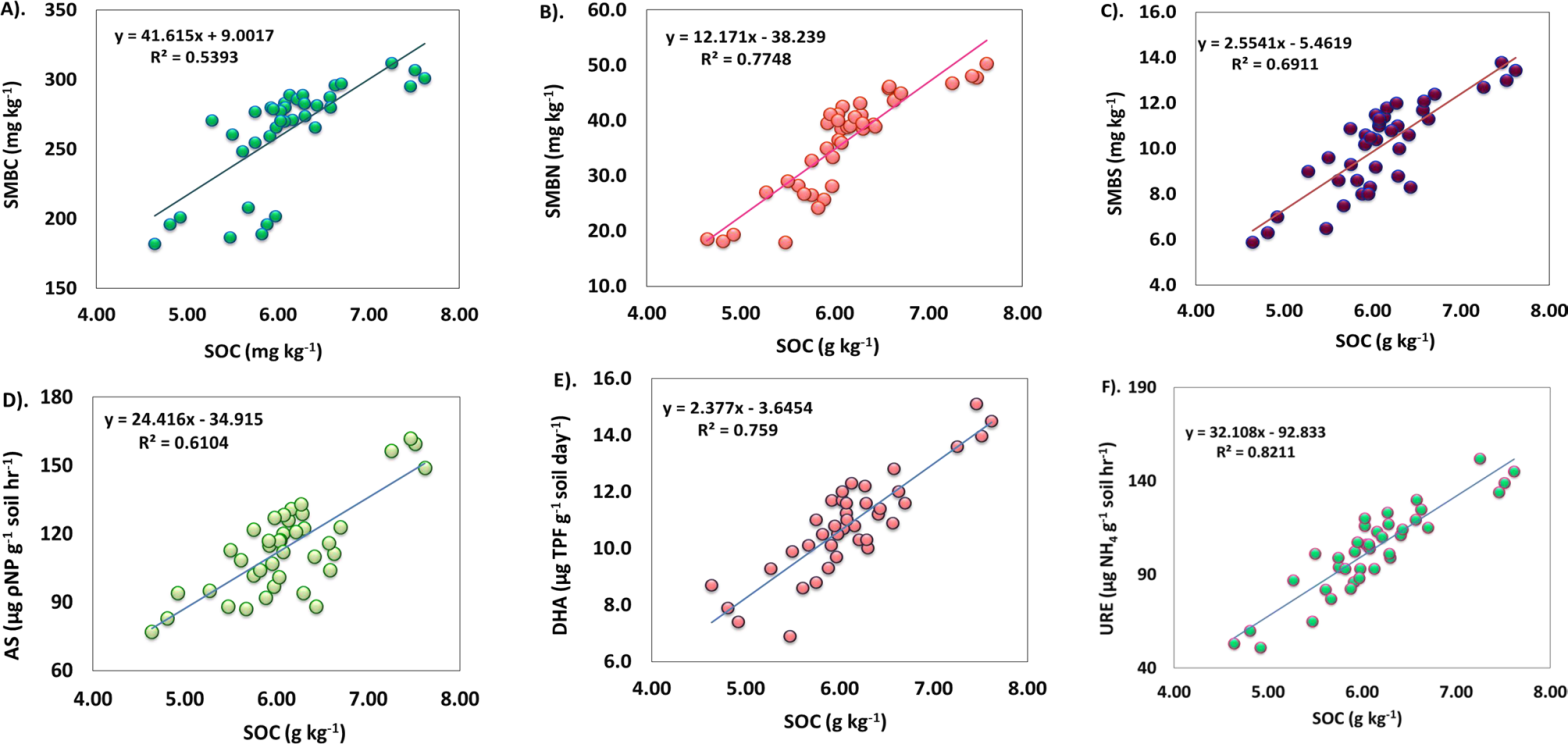
A simple linear regression model for long term fertilization and manuring on relationship between SOC, microbial biomass and soil enzymes in sandy clay loam soil. ^†^*SMBC* soil microbial biomass carbon, *SMBN* soil microbial biomass nitrogen, *SMBS* soil microbial biomass sulphur, *AS* aryl sulphatase, *DHA* dehydrogenase and *URE* urease.

**Figure 5:**
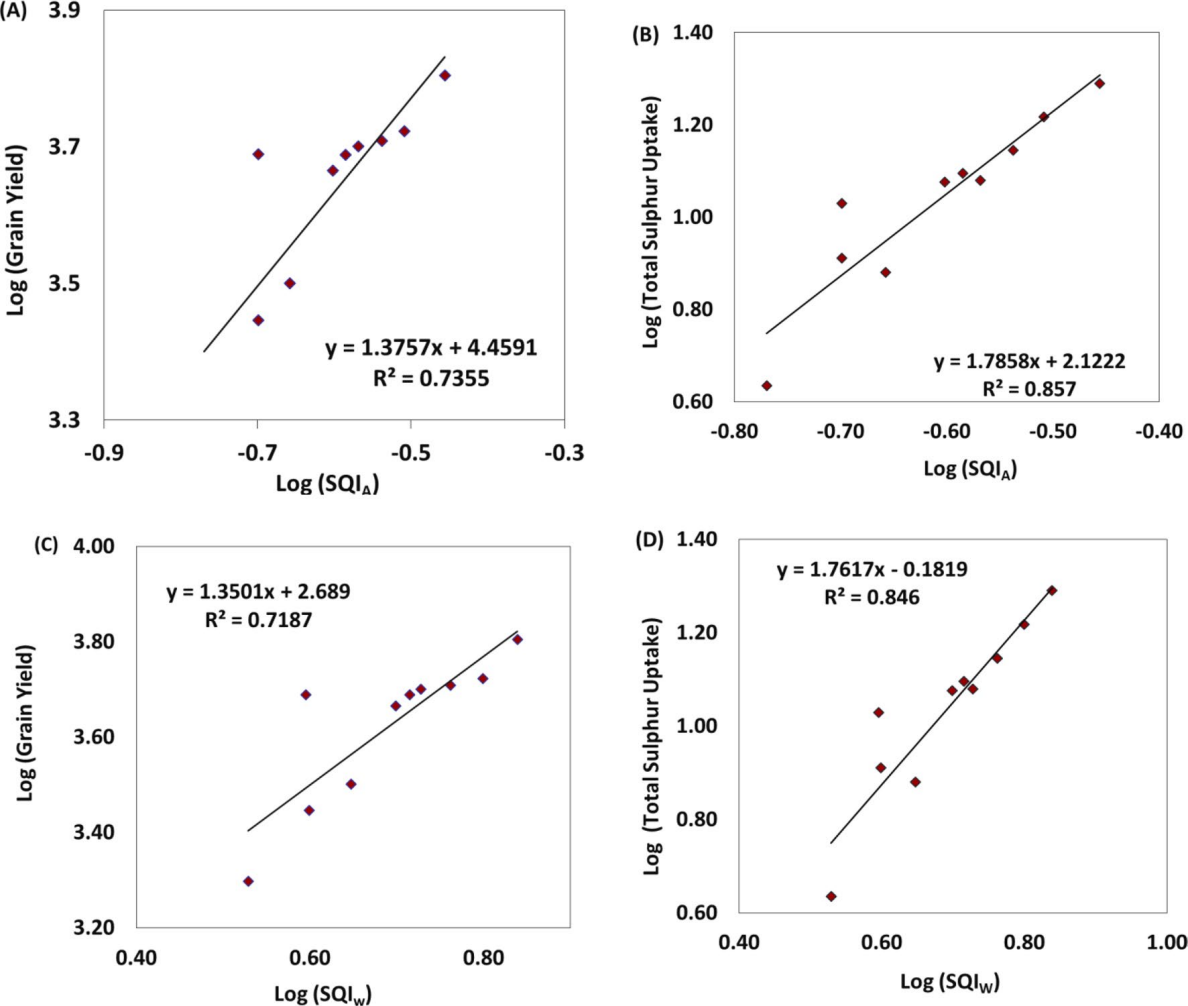
Validation of SQI with grain yield (kg ha^−1^) of maize and total sulphur uptake (kg ha^−1^) in an sandy clay loam soil (n = 10). *SQI_*W*_ Weighed Soil Quality Index, *SQI*_*A*_ Weighed Additive Soil Quality Index.

In addition, specific indicators for S transformation in these soils were identified, including SOC (up to 0.45 m), soil N (at three depths), SMBS (r^2^ = 0.93 and 0.91), SMBN (r^2^ = 0.97 and 0.92), SMBC (r^2^ = 0.96 and 0.86), and enzyme activities of AS (r^2^ = 0.85 and 0.84), DHA (r^2^ = 0.97 and 0.89), and URE (r^2^ = 0.98 and 0.93) (Fig. 4). These indicators played a crucial role in total S uptake and maize grain yield. Two distinct indices, the Weighted Additive and Additive indices (Supplementary Table 10), were developed to assess the impact of different treatments. The results indicated that the use of NPK + FYM contributed to better crop productivity, enhanced soil fertility, and S transformation (SQI_W_: 6.90, SQI_A_: 0.35), followed by 150% NPK (SQI_W_: 6.30, SQI_A_: 0.31) and 100% NPK + Zn (SQI_W_: 5.78, SQI_A_: 0.29). To validate the indices, a simple regression was employed, using grain yield and total S uptake of maize as the dependent variables (y) and SQIs as the independent variables (x) in the regression model (Fig. 5). The estimated regression coefficients were found to be statistically significant, with an r^2^ of 0.69 for SQI_A_ and 0.69 for SQI_W_ in relation to grain yield. Additionally, the r^2^ were 0.79 for SQI_A_ and 0.78 for SQI_W_ when examining the total sulphur uptake of maize in *Inceptisol*.

## Discussion

### Long term fertilization and intensive cropping on soil sulphur distribution, nexus on SOC and soil enzyme activities

In soil ecosystems, S dynamics are controlled by fertilisation practices and cropping system. Sulphur is an important nutrient instead the SO_4_^2−^–S is easily accessible by the plants. Regardless of the treatments, INM has significantly increased the soil available sulphur by application of SSP fertilizer, which has 16% S, and has a tendency to mineralize could be the cause of the increases in S^32^. Also, addition of FYM supplementation acted as a substrate for microbes which mineralize the applied fertilizer as well the plant biomass gave the additional nutrients to the plants. The plots that received DAP as a P source (S free) and the control plots had lowest SO_4_^2−^–S levels (Fig. 1). The exclusion of S from nutrient sources has the potential to degrade major and micronutrients over time and decrease soil microbial biomass, both of which are important for the mineralization of native soil^33^ and^34^). This outcome was consistent with the result of Lavanya et al.^35^, who found that treatments that received more single superphosphate^36^ had higher accessible sulphur contents in long-term fertilized soils under finger millet-maize cropping systems. The declining trend of SO_4_^2−^–S from the surface (0–0.15 m) to subsurface (0.3–0.45 m) soil may be attributed to the manual addition of fertilizers, manures, and other anthropogenic activities^37^.

Another easily mineralizable portion of sulphur is WSS and the application of S containing phosphatic fertilizers or manures increase the WSS in soil. A significant increase of WSS was registered in INM (NPK + FYM) than super optimal doses of nutrients (Fig. 1). These because of FYM act as a potential catalyst of S mineralization along with application of chemical fertilisers (SSP). Comparable results was found by Gourav et al.^38^ who stated that, combination of FYM with inorganic S addition lead to S mineralization by the soil microbial activity. Surface strata to sub surface the availability of water soluble sulphur got declined and the trend of availability was influenced by the treatments in each respective layers^39^ and^40^). The lowest water soluble sulphur was observed in absolute control (T_10_), S free plot (T_9_) and N alone (T_7_) plots. These might be due to elimination of S sources with intensive cropping could deteriorate the native S and disturb the equilibrium over the years and similar result was found by Lavanya et al.^35^. Similar results was reported by Suran et al.^41^.

One important form of organic sulphur is heat soluble sulphur, and it influences the soil's sulphur content (Fig. 1). The significant changes of HSS were noted in 100% NPK + FYM of 10 t ha^−1^ plot in each layer of soil from surface to subsurface strata^42^. The treatment with the greatest HSS content (100% NPK + FYM) also had the highest organic matter content, which might be attributed to the greater HSS content in this treatment. Other than NPK + FYM and NPK alone plots, the application of ZnSO_4_ along with NPK treated plots also have a greater influences of heat soluble sulphur may be due to the S contain fertilisers and manures^43^. The lowest HSS was noticed in absolute control, S free plot and N alone treatments because of exclusion of S fertilizer over five decades. The accountability of HSS was higher than SO_4_^2−^–S and WSS in in each respective layer. Similarly, Basumatary and Das^44^ who reported, during heat treatment, the HSS has higher than available S and water soluble fraction. According to the results, the HSS content was declined from surface to sub surface strata because of low organic matter and less microbial activity in soil^45^.

Among the various fraction of S, OS contributed more S nearly 80–90% in agricultural soils (Fig. 1). A considerable increase in organic S was seen in the treatment that received graded dosages of nutrients ranging from 50 to 150% NPK. This rise might be due to the addition of sulphur by the use of P fertilizer^38,46,47^. The organic S content has declined from the surface to the subsurface, attributed to the diminished distribution of nutrients resulting from the application of manure and fertilizer in the soil^48^. The lowest organic sulphur was observed in absolute control, N alone and 100% NPK (–S free) plots in long run. Continuous application of FYM could add the plant available nutrients so that the microbial biomass has increased in soil. Microbial biomass has the capacity to store nutrients when their concentrations are high, and it can subsequently supply these nutrients to plants when the organic carbon content becomes depleted in the soil^49^. Similarly, Yang et al.^50^ noted that application of FYM has increase the organic S accumulation in soil. Furthermore, Das et al.^51^ noted that OS in soil exhibits a positive correlation with all forms of sulphur, indicating a direct reflection of the dynamic equilibrium among them.

The total sulphur in agricultural soils depends on primary minerals, organic compounds, sorbed S, solution S, farming practices and cropping pattern. Continuous addition of fertiliser, manure and intensive cropping can altered the soil S dynamics and its equilibrium of soil (Fig. 1). Despite that, significant increase of total sulphur was observed in NPK + FYM plots followed by super optimal doses than recommended dose and 100% NPK + Zn plots in soil. Application of chemical fertiliser nutrients as well organic amendments could build up the soil S reserves over the years of cultivation^52^. Similarly, Kumar et al.^53^ who revealed, the greater fertility enhances the total and available S content by addition of SSP and S application via organic manures. The TS content was reduced from surface to subsurface layers and lowest TS content in absolute control plots^54^. This might due to the continual cropping without replenishing sulphur in the soil causes sulphur to be released from other pools into the available pool for crop absorption and in long run the equilibrium got disturbed^35,55^). Additionally, Elkin et al.^56^ have corroborated that due to low inputs, soil organic pools are depleted, resulting in diminished responsiveness to sulphur fertilization in crops due to reduced soil organic matter^57^.

SOC has the ability to act as a stimulant for nutrient availability during plant growth by means of soil mineralization. The increase of SOC exerts a positive influence on various physical, chemical, and biological properties, as they are all interdependent in soil.

The SOC considerably increases throughout the course of five decades (4.96 to 7.46 g kg^−1^ in 2022) since the experiment's inception (3.0 g kg^−1^ in 1972), despite the practise of varied nutrition management measures (Fig. 2 and Supplementary Table 7). Also, the SOC of 7.46 g kg^−1^ can be increased with continuous use of NPK with FYM 10 t ha^−1^, followed by super optimum dosages (6.62 g kg^−1^). The increase in SOC within a nutrient cycle system depends on the input (addition) and output (rate of decomposition) rates of organic matter. In INM, the rate of input and output has been maintained by the application of manures, which supply recalcitrant C^58^ and a quantity of nutrients to the soil, and applied fertilizers would support the plants for production of root and plant biomass^59^. Similarly, Liu et al.^60^ who revealed that the addition of carbon through plant biomass, high humification rate constants and a lower decay rate in all fertilized plots, including control treatments, have all expedited the rate of increase of SOC. Regardless of the nutrient management practices, the SOC status shown to decrease with soil depth. The beneficial effects of fertilization were mostly limited to the surface layers. This also implies that SOC was strongly correlated with root C inputs, FYM, and crop residues, which led to its build up in surface soils. These findings confirmed those of Liang et al.^61^, who discovered that under the wheat–maize cropping system, SOC in FYM amended plots decreased with soil depth of organic and inorganic fertilization.

In addition to transforming soil organic matter (SOM) and other insoluble substances, soil microbial biomass is an early indicator of soil quality and is essential to the cycling of soil N, P, and S nutrients. The significant increase of SMBC, SMBN and SMBS are observed in NPK plus FYM than super optimal dose (150%NPK). The increase in soil microbial biomass containing C, N and S can be attributed to the application of both fertilizer FYM which provide nutrients and substrates essential for microbial growth and multiplication^62^, compared to plots treated solely with fertilizer. The rate of increase of fertiliser dose from 50% NPK to 150% NPK dose could raise the SMB which might have ascribed to addition of SOM (residues, plant litters and root biomass) and microbial growth^63^. Omission of major nutrients especially P and K (N alone) decreases the SMB^64^ by affect the soil buffering capacity which directly influenced available nutrients, SOC^65^ and microbial composition^66^ of the soil. There was less positive correlation with absolute control (T_10_) and accumulation of SMB (C, N and S) due to low SOC, enzyme activities (DHA, AS and URE) in soil^67^ (Fig. 3).

Enzymes, which can be continually synthesized and stored in soil, are instances of so-called protein molecules that can facilitate the vital function of regulating the energy and matter in an ecosystem. The hydrolytic and oxidative enzyme activities in soil are the important soil quality proxy that catalyse the transformation of nutrients^68^.

The reactions of urea with hydroxyurea, dihydroxyurea, and semicarbazide can be catalysed by the extracellular enzyme urease (urea amidohydrolase). It uses urea as a substrate as well, converting it to ammonia and CO_2_^24,69,70^. Regardless of the different nutrient management techniques, using NPK fertilizers in addition to FYM 10 t ha^−1^ would increase URE activities^71^ more than using mineral fertilizers alone at super optimum levels. Because FYM addition can provide the energy substrate for the microbiomes and nutrients to the plants, chemical fertilization could decrease soil fertility by less accumulation of SOC, SMB (C and N), TN^72,73^ and buffering capacity^74^.

An oxidoreductase enzyme called DHA catalyzes the oxidation of the substrate in soil and is a representative of the microflora's total oxidative activity^75^. Plots treated with N will have less of an effect, and DHA activities will increase with the addition of NPK with FYM 10 t ha^−1^, followed by treatments with mineral fertilizers. The increase in DHA activities may have been influenced by the accumulation of soil microbial biomass (C and N), which is a labile pool of SOC that may be utilized by plants and microorganisms^76–78^. Plots that were fertilized with N alone and control plots had lower DHA levels due to the decline in soil fertility^79^.

In order to hydrolyze ester sulphate into SO_4_^2−^ form, an enzyme known as aryl sulphatase (AS) dissolves the O-S bond in soil. Higher AS activities in NPK + FYM, 150% NPK and 100% NPK + Zn were found in the soil. This may be due to higher Zn levels^80^ and recalcitrant humus stabilised an enzyme that stops further microbial breakdown in soil, which might have been caused by the addition of SOM^81^, which provided a substrate for bacteria. Other recommended dosages, such as 100% NPK, 100% NPK + HW, and 100% NP, are comparable to one another. This may be because SSP, used as a P source and a source of S, adds S to the soil while also providing the nutrients needed to maintain AS activities in the soil^82^. Also, the notable decrease in the AS activity in the sulphur free plot, 100% N alone and control plots which may be the lack of sulphur fertilization could affect the microorganism’s cellular development and thereby the activity of AS has decreased in soil^83^.

Regardless of the nutrient management techniques, 100% NPK + FYM of 10 t ha^−1^ and super optimum dosages plots showed an improvement in maize grain and straw yield^38,41^. The application of FYM could have given nutrients and SOM substrate to the soil, improving the soil rhizosphere, which was ascribed to the increase in biomass and yield. In the root zone environment, the rhizosphere alteration improves nutrient availability by lowering the volatilization of NH_4_^+^, leaching of NO_3_^−^, solubilisation of P, oxidation of S, and chelation of metals with humus. Continuous N fertilization had an adverse impact on yield and control when sandy clay loam soil were utilized under intensive cropping.

**Figure 6:**
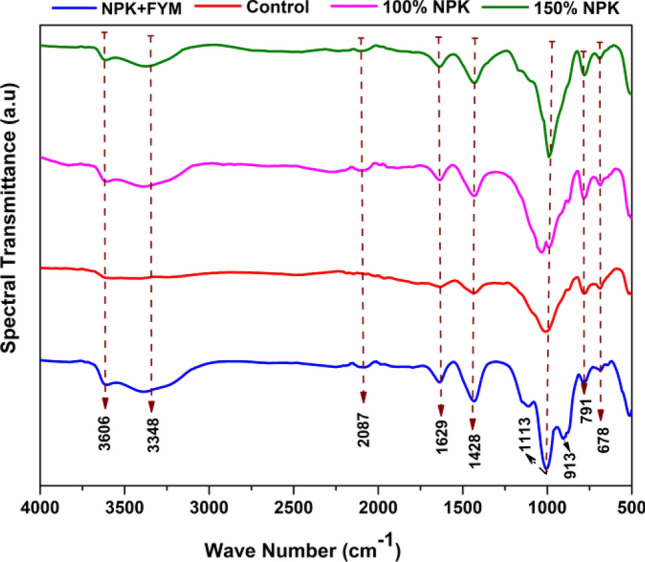
Characterization of SOC functional groups by FT–IR spectroscopy.

FT-IR spectroscopy serves to describe the characters of SOC, and variations in spectral transmittance are suggestive of different functional groups that are present in the soil (Fig. 6) Functional groups like OH, C–O, C–H, and S–C–N are typically present in agricultural soils. This is mostly because plant and root biomass contributes to a linear rise in SOC content. Fertilizer application, either by itself or in conjunction with manure, has the potential to modify the SOC composition, which in turn affects the biochemical characteristics of SMBN (r^2^ of 0.77) and SMBS (r^2^ of 0.69).

The state of SOM provides insights into the functional properties of SOC. Notably, the presence of polyphenol groups like acetyl and sulfoxide (1428–1414 cm^−1^), imine/oxime, and ketones (1628–1608 cm^−1^) is observed in plots with mineral fertilizer application and integrated nutrient management (INM) plots. This suggests that fertilized or manure-amended soils contain aromatic carbonyl and carboxylate functional groups, which contribute to stabilizing SOC against microbial decomposition. This is consistent with the findings of Zibilske and Materon^84^, who found that the incorporation of high-polyphenolic content from cotton residues was the cause of the existence of protein-rich material and organic nitrates in the 1660 to 1630 cm^−1^ band. Additionally, Heller et al.^85^ noted out that a high C–H:C=O ratio could protect the C–H-containing SOM from microbial degradation because heavy metals, particularly Fe, adsorb to it.

Thiocyanate functional groups are present at 2102 to 2075 cm^−1^ in all four treatments, representing organosulfur functional groups that catalyze the oxidation of organic carbon with the enzyme sulfur transferase. The addition of fertilizers, with or without manure, increases SOC content, which correlates with enhanced enzyme activities of AS (r^2^ of 0.61), DHA (r^2^ of 0.76), and URE (r^2^ of 0.82) over a five-decade span on *Inceptisol*.

The transmittance spectra in the 995–699 cm^−1^ region correspond to mostly monosubstituted aromatic C–H and C=C bonds. As the wavenumber increases, the degree of substitution may also rise. These data are consistent with work by Calderón et al.^86^, which reported that the 700 and 975 cm^−1^ range can suggest the existence of aromatic CH groups in bluestem soils, but that accurate identification may be difficult due to the possibility that it includes a combination of organic and mineral functional groups^87^.

**Figure 7:**
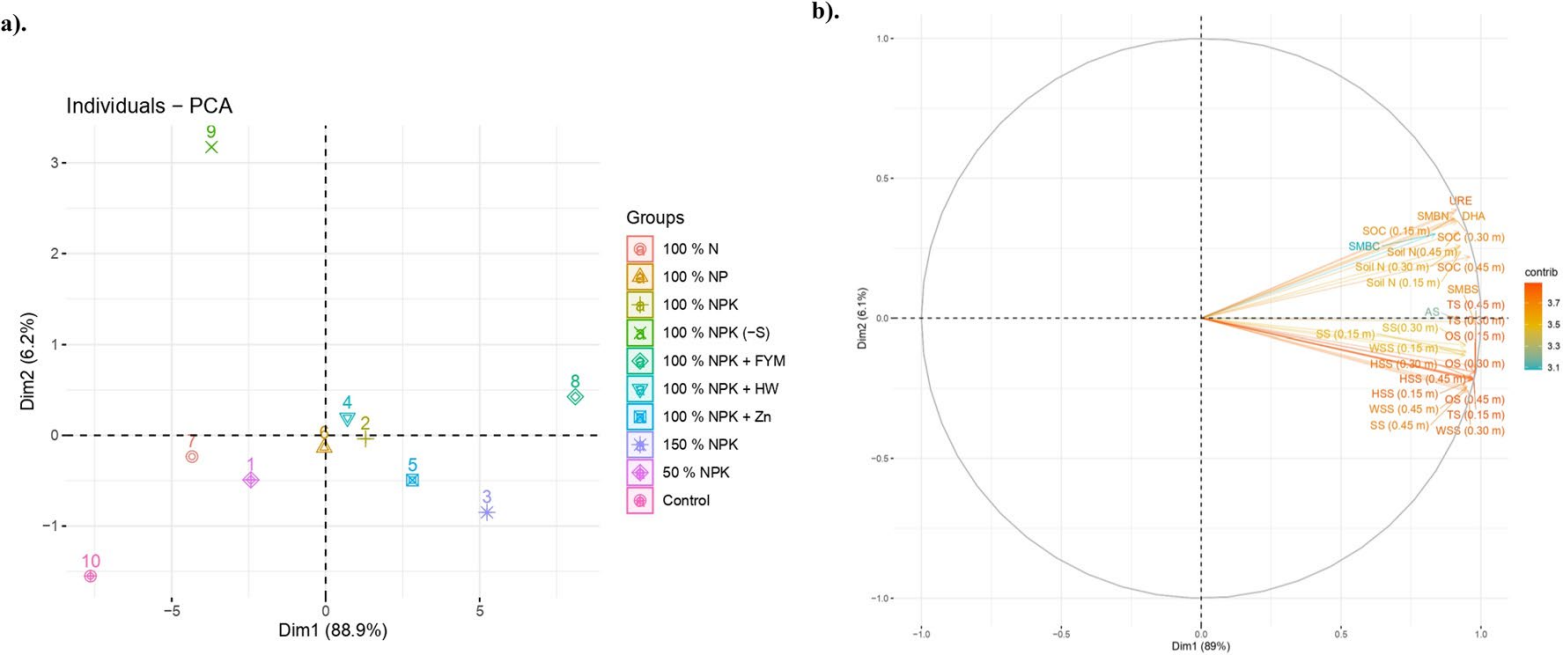
(**a**) Individuals-PCA-biplot depicting the contribution of different nutrient management practices*.* (**b**) Biplot depicting the contribution of soil indicators towards different nutrient management practices in an *Inceptisol.*

For the development of soil quality indices through PCA, it was demonstrated that the application of various nutrient management practices could affect soil sulphur transformation and SOC, both of which significantly influence maize crop uptake and grain yield. In biplot axes showed, the quadrant 1 (upper right) have critical indicator for S transformations are SOC (upto 0.45 m), soil biochemical properties (SMBN, SMBC and SMBS) and enzyme activities (URE, DHA and AS) (Fig. 7). SOM oxidation is directly linked with soil S and which interfere the aggregate formation, stability of aggregates, moisture retention, nutrient conservation and acts as buffer for thermal stability etc.,^88,89^. SOC is a potential indicator for soil biological activities and diversity^90^ and soil enzymes drives the major nutrient transformation in soil. Soil amended with manure and fertilizers significantly increase the ureases activity^91^ also it linearly increase with increases of soil N and SOC^92^. For the oxidation of SOM, the enzyme DHA is oxidized^93^ and transfers the H^+^ ions from organic substrate to inorganic acceptors, which might boost the enzyme activities that increase the buildup of ammonifiers in soil. For S cycling, the organic sulfate esters are hydrolyzed into phenols and sulfate^94^ and application of inorganic fertilizers could reduce the AS activity but amended with manure could increase the AS activity. Nevertheless, the nutrient cycling and SOM accumulation are linearly correlated with MBC which reflects the soil fertility and soil quality^95^. Our results revealed that, continuous use of manure plus inorganic fertilizers (T_8_) would increase the SOC accumulation, microbial biomass C, N and S also enzyme activities (URE, DHA and AS) in sandy clay loam calcareous soil. Next to that, in quadrant 2 had the S fractions of WSS, HSS, OS, SS and TS with different specified depths (upto 0.45 m). Regardless of the different nutrient management practices, NPK along with FYM increased the all forms of S (SS, WSS, HSS, OS and TS) followed by super optimal doses (150% NPK) plot. With increase of the fertilizer doses from 50% NPK to 150% NPK has increased the S fractions (Fig. 7). And continuous application of single nutrient or nitrogenous fertilizers could deteriorate the soil quality and sustainability. Similar results was reported by Bappa Das et al.^96^ under rice–wheat cropping system. According to the Nisab et al.^97^ revealed that, WSS was significantly correlated with HSS ad SS in lateritic soils of eastern India.

## Conclusions

The present investigation has revealed that comprehensive insights into soil sulphur distribution, alongside soil N, SOC, microbial biomass, and enzymes, are most effectively obtained through long-term fertilizer experiments conducted in intensive cropping systems over successive years. Sulphur species notably accumulated at the surface strata but showed a decreasing trend with increasing soil depth. Regardless of the applied treatments, the most significant increase in sulphur fraction occurred with the use of mineral fertilizers (100% NPK) supplemented with 10 t ha^−1^ of FYM, followed by 150% NPK and 100% NPK + Zn. The hierarchy of sulphur species accountability in these experiments is as follows: Organic sulphur > Heat-soluble sulphur > SO_4_^2−^–S > Water-soluble sulphur. Omitting sulphur nutrients, especially in plots with absolute control, 100% N alone, and 100% NPK (with DAP, as a P source), resulted in a decline in soil sulphur levels. Key soil factors such as SOC, soil N, microbial biomass (C and N), and enzyme activities (URE, DHA, and AS) play crucial roles as catalysts for sulphur transformation. Following soil nitrogen, sulphur emerges as a vital nutrient for enhancing crop productivity, and the judicious use of fertilizers, along with organic amendments like FYM, significantly contributes to increased maize production in the context of long-term agricultural goals.

Looking ahead to future research, a crucial focus should be on understanding the rate of sulphur mineralization, discerning how much sulphur is taken up from fertilisers and how much undergoes mineralization, with a specific emphasis on elucidating the solubilisation processes mediated by microorganisms. In-depth knowledge of microbial communities can be achieved through metagenomics, enabling the identification of predominant microbes involved in biogeochemical cycling. This microbial insight will be instrumental in unravelling the intricate mechanisms governing sulphur dynamics in the soil and will pave the way for more informed and sustainable agricultural practices to address the challenges of long-term soil health and crop productivity.

### Supplementary Information

Supplementary Tables.

## Data Availability

All data generated or analysed during this study are included in this published article and its supplementary information file (Supplementary Table from 1 to 10). The data that support the findings of this study are available from the corresponding author and the same will be provided upon reasonable request.
