# Author Correction: Long-term fertilization and manuring effects on the nexus between sulphur distribution and SOC in an *Inceptisol* over five decades under a finger millet–maize cropping system

**DOI:** 10.1038/s41598-024-63390-4

**Published:** 2024-06-03

**Authors:** B. Gokila, G. Manimaran, D. Jayanthi, K. Sivakumar, G. Sridevi, S. Thenmozhi, M. Elayarajan, A. Renukadevi, R. Sudha, P. Balasubramanian

**Affiliations:** 1 Department of Soil Science & Agrl. Chemistry, Tamil Nadu Agricultural University, Coimbatore, 641 003 India; 2 Department of Agricultural Economics, Tamil Nadu Agricultural University, Coimbatore, 641 003 India; 3 Department of Agronomy, Tamil Nadu Agricultural University, Coimbatore, 641 003 India

Correction to: *Scientific Reports* 10.1038/s41598-024-60357-3, published online 29 April 2024

The original version of this Article contained a repeated error in the Affiliations, where ‘Tamil Nadu Agricultural University’ was incorrectly given as ‘Tamil Nadu Agricultural Chemistry’.

The correct affiliations are listed below.

Department of Soil Science & Agrl. Chemistry, Tamil Nadu Agricultural University, Coimbatore, 641 003, India

Department of Agricultural Economics, Tamil Nadu Agricultural University, Coimbatore, 641 003, India

Department of Agronomy, Tamil Nadu Agricultural University, Coimbatore, 641 003, India

The original Article has been corrected.

