# Supplementary material for: Long-term fertilization and manuring effects on the nexus between sulphur distribution and SOC in an Inceptisol over five decades under a finger millet–maize cropping system
Source: Sci Rep. 2024 Apr 29;14:9758. doi: 10.1038/s41598-024-60357-3 (PMC11058816; doi:10.1038/s41598-024-60357-3)
Supplement: Supplementary file 1 — Supplementary Tables. [file 41598_2024_60357_MOESM1_ESM.docx]

**Long-term fertilization and manuring effects on the nexus between sulphur distribution and SOC in an *Inceptisol* over five decades under a finger millet-maize cropping system**

*Gokila.B, Manimaran.G, Jayanthi.D, Sivakumar.K, Sridevi.G, Thenmozhi.S, Elayarajan.M, Renukadevi. A, Sudha.R and Balasubramanian.P

**Table.1 Effect of continuous fertilisation and cropping on SO_4_^2-^ - S (mg kg^-1^) in sandy clay loam soil**

| ***Treatments*** | **SO_4_^2^ - S (mg kg^-1^)** | | |
| --- | --- | --- | --- |
|  | **0 - 15 cm** | **15 - 30 cm** | **30 - 45 cm** |
| 50% NPK | 13.7*^d^* (±0.3) | 8.5*^de^* (±0.4) | 7.3*^d^* (±0.4) |
| 100% NPK | 17.1*^c^* (±0.6) | 11.7*^bc^* (±0.5) | 9.8*^bc^* (±0.5) |
| 150% NPK | 34.4*^a^* (±0.5) | 22.7*^a^* (±0.7) | 11.1*^ab^* (±0.6) |
| 100% NPK + HW | 16.7*^c^* (±0.8) | 11.3*^bc^* (±0.7) | 9.3*^bc^* (±0.4) |
| 100% NPK + Zn | 20.1*^b^* (±0.5) | 13.1*^b^* (±0.6) | 10.6*^ab^* (±0.5) |
| 100% NP | 16.5*^cd^* (±0.6) | 10.7*^cd^* (±0.7) | 8.5*^cd^* (±0.4) |
| 100% N | 10.6*^e^* (±0.6) | 7.2*^ef^* (±0.4) | 5.0*^e^* (±0.3) |
| 100% NPK + FYM | 35.8*^a^* (±1.0) | 23.6*^a^* (±0.5) | 12.1*^a^* (±0.3) |
| 100% NPK (-S) | 8.4*^ef^* (±0.4) | 6.2*^f^* (±0.4) | 4.2*^e^* (±0.3) |
| Control | 7.0*^f^* (±0.4) | 5.3*^f^* (±0.3) | 3.8*^e^* (±0.2) |

***** In each column, values followed by the common letters are not significantly different at P ≤ 0.05 based on the Tukeys HSD and values followed by ± symbol denotes the standard error of means.***

**Table.2 Effect of continuous fertilisation and cropping on WSS (mg kg^-1^) in a sandy clay loam soil**

| ***Treatments*** | **WSS (mg kg^-1^)** | | |
| --- | --- | --- | --- |
|  | **0 - 15 cm** | **15 - 30 cm** | **30 - 45 cm** |
| 50% NPK | 7.37*^cde^* (±0.50) | 4.91*^c^* (±0.60) | 2.53*^d^* (±0.22) |
| 100% NPK | 8.83*^c^* (±0.52) | 6.37*^bc^* (±1.01) | 3.44*^bc^* (±0.25) |
| 150% NPK | 15.1*^a^* (±0.49) | 8.94*^a^* (±0.94) | 4.17*^b^* (±0.26) |
| 100% NPK + HW | 8.22*^cd^* (±0.52) | 6.06*^c^* (±0.96) | 3.11*^cd^* (±0.17) |
| 100% NPK + Zn | 11.9*^b^* (±0.60) | 7.94*^ab^* (±0.84) | 3.51*^bc^*(±0.21) |
| 100% NP | 8.26*^cd^* (±0.46) | 5.64*^c^* (±0.64) | 3.09*^cd^*(±0.19) |
| 100% N | 6.55*^de^* (±0.35) | 2.63*^d^* (±0.43) | 1.50*^e^*(±0.20) |
| 100% NPK + FYM | 15.5*^a^* (±0.52) | 9.25*^a^* (±0.83) | 5.18*^a^*(±0.13) |
| 100% NPK (-S) | 5.93*^e^* (±0.32) | 2.07*^d^* (±0.38) | 0.56*^f^*(±0.04) |
| Control | 5.66*^e^* (±0.40) | 1.98*^d^* (±0.48) | 0.27*^f^*(±0.02) |

***** In each column, values followed by the common letters are not significantly different at P ≤ 0.05 based on the Tukeys HSD and values followed by ± symbol denotes the standard error of means.***

**Table.3 Effect of continuous fertilisation and cropping on HSS (mg kg^-1^) in a sandy clay loam soil**

| ***Treatments*** | **HSS (mg kg^-1^)** | | |
| --- | --- | --- | --- |
|  | **0 - 15 cm** | **15 - 30 cm** | **30 - 45 cm** |
| 50% NPK | 31.6*^de^* (±1.3) | 18.9*^d^* (±0.53) | 7.9*^ef^* (±0.55) |
| 100% NPK | 38.2*^bc^* (±1.2) | 25.6*^c^* (±0.52) | 10.7*^cd^* (±0.51) |
| 150% NPK | 42.4*^ab^* (±0.8) | 32.2*^ab^* (±0.69) | 13.5*^ab^* (±0.48) |
| 100% NPK + HW | 35.3*^cd^* (±0.8) | 23.6*^c^* (±0.54) | 10.5*^d^* (±0.46) |
| 100% NPK + Zn | 40.1*^b^* (±0.7) | 30.2*^b^* (±0.87) | 12.2*^bc^* (±0.41) |
| 100% NP | 34.7*^cd^* (±0.9) | 23.4*^c^* (±0.56) | 9.5*^de^* (±0.47) |
| 100% N | 28.0*^ef^* (±0.6) | 19.2*^d^* (±0.68) | 7.2*^f^* (±0.40) |
| 100% NPK + FYM | 45.5*^a^* (±0.8) | 33.7*^a^* (±0.71) | 14.8*^a^* (±0.34) |
| 100% NPK (-S) | 24.6*^fg^* (±0.5) | 14.8*^e^* (±0.51) | 5.1*^g^* (±0.23) |
| Control | 22.8*^g^* (±0.5) | 13.9*^e^* (±0.50) | 4.6*^g^* (±0.27) |

***** In each column, values followed by the common letters are not significantly different at P ≤ 0.05 based on the Tukeys HSD and values followed by ± symbol denotes the standard error of means.***

**Table.4 Effect of continuous fertilisation and cropping on Organic Sulphur (mg kg^-1^) in a sandy clay loam soil**

| ***Treatments*** | **OS (mg kg^-1^)** | | |
| --- | --- | --- | --- |
|  | **0 - 15 cm** | **15 - 30 cm** | **30 - 45 cm** |
| 50% NPK | 198*^e^* (±7) | 173*^f^* (±4.2) | 123*^e^* (±3.9) |
| 100% NPK | 235*^cd^* (±4) | 216*^cd^* (±4.6) | 148*^d^* (±4.3) |
| 150% NPK | 273*^b^* (±4) | 265*^b^* (±4.1) | 192*^b^* (±3.6) |
| 100% NPK + HW | 226*^d^* (±3) | 209*^de^* (±4.7) | 144*^d^* (±3.5) |
| 100% NPK + Zn | 248*^c^* (±5) | 225*^c^* (±3.8) | 171*^c^* (±3.2) |
| 100% NP | 221*^d^* (±4) | 201*^e^* (±3.7) | 139*^d^* (±2.8) |
| 100% N | 182*^e^* (±4) | 148*^g^* (±2.9) | 97*^f^* (±3.0) |
| 100% NPK + FYM | 303*^a^* (±5) | 283*^a^* (±3.5) | 215*^a^* (±3.2) |
| 100% NPK (-S) | 157*^f^* (±3) | 123*^h^* (±2.8) | 80*^g^* (±3.0) |
| Control | 149*^f^* (±4) | 127*^h^* (±2.8) | 77*^g^* (±3.4) |

***** In each column, values followed by the common letters are not significantly different at P ≤ 0.05 based on the Tukeys HSD and values followed by ± symbol denotes the standard error of means.***

**Table.5 Effect of continuous fertilisation and cropping on Total Sulphur (mg kg^-1^) in a sandy clay loam soil**

| ***Treatments*** | **TS (mg kg^-1^)** | | |
| --- | --- | --- | --- |
|  | **0 - 15 cm** | **15 - 30 cm** | **30 - 45 cm** |
| 50% NPK | 242*^d^* (±4.0) | 173*^f^* (±3.6) | 117*^e^* (±3.8) |
| 100% NPK | 287*^c^* (±5.6) | 217*^d^* (±3.9) | 150*^d^* (±2.6) |
| 150% NPK | 344*^a^* (±4.9) | 269*^b^* (±3.4) | 195*^b^* (±3.4) |
| 100% NPK + HW | 275*^c^* (±4.7) | 215*^d^* (±3.5) | 146*^d^* (±3.4) |
| 100% NPK + Zn | 310*^b^* (±4.2) | 240*^c^* (±3.6) | 173*^c^* (±3.2) |
| 100% NP | 269*^c^* (±4.6) | 195*^e^* (±3.6) | 144*^d^* (±2.7) |
| 100% N | 207*^e^* (±3.0) | 147*^g^* (±3.3) | 95*^f^* (±2.6) |
| 100% NPK + FYM | 357*^a^* (±3.1) | 315*^a^* (±3.4) | 213*^a^* (±3.2) |
| 100% NPK (-S) | 193*^e^* (±3.9) | 127*^h^* (±2.7) | 85*^g^* (±2.8) |
| Control | 188*^e^* (±5.8) | 122*^h^* (±2.8) | 78*^g^* (±2.9) |

***** In each column, values followed by the common letters are not significantly different at P ≤ 0.05 based on the Tukeys HSD and values followed by ± symbol denotes the standard error of means.***

**Table.6 Effect of continuous fertilisation and cropping on SOC (g kg^-1^) in sandy clay loam soil**

| ***Treatments*** | **SOC (g kg^-1^)** | | |
| --- | --- | --- | --- |
|  | **0 - 15 cm** | **15 - 30 cm** | **30 - 45 cm** |
| 50% NPK | 5.53*^d^* (±0.10) | 4.71*^cd^* (±0.11) | 4.32*^cde^* (±0.11) |
| 100% NPK | 6.07*^c^* (±0.05) | 5.22 *^bc^* (±0.14) | 4.68*^abcd^* (±0.11) |
| 150% NPK | 6.62*^b^* (±0.03) | 5.79 *^ab^* (±0.17) | 5.23*^ab^* (±0.10) |
| 100% NPK + HW | 6.15*^c^* (±0.06) | 5.28*^bc^* (±0.16) | 4.71*^abcd^* (±0.12) |
| 100% NPK + Zn | 6.26*^bc^* (±0.08) | 5.35 *^bc^* (±0.15) | 4.92*^abc^* (±0.12) |
| 100% NP | 5.93*^cd^* (±0.07) | 5.05 *^bc^* (±0.12) | 4.56*^cd^* (±0.18) |
| 100% N | 5.84*^cd^* (±0.06) | 4.97 *^bc^* (±0.11) | 4.19*^de^* (±0.07) |
| 100% NPK + FYM | 7.46*^a^* (±0.08) | 6.55 *^a^* (±0.24) | 5.28*^a^* (±0.18) |
| 100% NPK (-S) | 6.18*^c^* (±0.11) | 5.31*^bc^* (±0.26) | 4.62*^bcd^* (±0.19) |
| Control | 4.96*^e^* (±0.18) | 4.05 *^d^* (±0.16) | 3.80*^a^* (±0.09) |

***** In each column, values followed by the common letters are not significantly different at P ≤ 0.05 based on the Tukeys HSD and values followed by ± symbol denotes the standard error of means.***

**Table.7 Effect of continuous fertilisation and cropping on KMnO_4_ - N (kg ha^-1^) in a sandy clay loam soil**

| ***Treatments*** | **KMnO_4_ - N (kg ha^-1^)** | | |
| --- | --- | --- | --- |
|  | **0 - 15 cm** | **15 - 30 cm** | **30 - 45 cm** |
| 50% NPK | 170*^ef^* (±2.1) | 135*^gh^* (±2.8) | 129*^de^* (±3.0) |
| 100% NPK | 190*^d^* (±1.8) | 169*^bc^* (±3.0) | 156*^b^* (±2.2) |
| 150% NPK | 233*^ab^* (±3.2) | 181*^b^* (±2.7) | 177*^a^* (±3.0) |
| 100% NPK + HW | 202*^cd^* (±3.0) | 154*^def^* (±2.7) | 152*^bc^* (±2.6) |
| 100% NPK + Zn | 214*^bc^* (±4.9) | 163*^cd^* (±3.1) | 160*^b^* (±2.3) |
| 100% NP | 194*^cd^* (±4.9) | 145*^fg^* (±3.2) | 141*^cd^* (±2.7) |
| 100% N | 186*^de^* (±3.1) | 148*^ef^* (±4.0) | 124*^e^* (±2.3) |
| 100% NPK + FYM | 243*^a^* (±3.5) | 211*^a^* (±3.0) | 181*^a^* (±2.7) |
| 100% NPK (-S) | 197*^cd^* (±6.2) | 159*^cde^* (±2.5) | 155*^b^* (±2.8) |
| Control | 158*^f^* (±3.8) | 123*^h^* (±2.0) | 117*^e^* (±2.9) |

***** In each column, values followed by the common letters are not significantly different at P ≤ 0.05 based on the Tukeys HSD and values followed by ± symbol denotes the standard error of means.***

**Table 8. Descriptive Analysis and Total Variance Explained by Principal Component Analysis (PCA)**

| **Total Variance Explained by PCA** | | | | | | | |
| --- | --- | --- | --- | --- | --- | --- | --- |
| **Component** | **Initial Eigenvalues** | | |  | **Extraction Sums of Squared Loadings** | | |
|  | **Total** | **% of Variance** | **Cumulative %** | **Total** | **% of Variance** | **Cumulative %** | **Weighed Factor** |
| 1. | 25.692 | 88.593 | 88.593 | 25.692 | 88.593 | 88.593 | 0.48 |
| 2. | 1.816 | 6.261 | 94.854 | 1.816 | 6.261 | 94.854 | 0.03 |
| **Descriptive Statistics** | | | **Component Matrix** | |  |  |  |
| **Indicators** | **Mean** | **SD** | **PC1** | **PC2** |  |  |  |
| SS (0-15) | 18.03 | 9.89 | 0.943 | -0.160 |  |  |  |
| SS (15-30) | 12.03 | 6.38 | 0.940 | -0.140 |  |  |  |
| SS (30-45) | 8.17 | 2.97 | 0.944 | -0.260 |  |  |  |
| WSS (0-15) | 9.32 | 3.61 | 0.937 | -0.175 |  |  |  |
| WSS (15-30) | 5.57 | 2.70 | **0.951** | -0.277 |  |  |  |
| WSS (30-45) | 2.73 | 1.55 | 0.947 | -0.252 |  |  |  |
| HSS (15-30) | 34.32 | 7.55 | **0.965** | -0.233 |  |  |  |
| HSS (15-30) | 23.55 | 6.96 | 0.957 | -0.238 |  |  |  |
| HSS (30-45) | 9.60 | 3.41 | 0.966 | -0.238 |  |  |  |
| OS (0-15) | 219 | 49.0 | **0.974** | -0.215 |  |  |  |
| OS (15-30) | 197 | 54.4 | **0.969** | -0.237 |  |  |  |
| OS (30-45) | 138 | 46.0 | **0.970** | -0.239 |  |  |  |
| TS (0-15) | 267 | 59.8 | **0.968** | -0.241 |  |  |  |
| TS (15-30) | 202 | 62.3 | **0.975** | -0.199 |  |  |  |
| TS (30-45) | 139 | 45.9 | **0.975** | -0.213 |  |  |  |
| SOC (0-15) | 6.1 | 0.65 | 0.911 | **0.322** |  |  |  |
| SOC (15-30) | 5.22 | 0.65 | 0.922 | **0.314** |  |  |  |
| SOC (30-45) | 4.63 | 0.45 | **0.965** | 0.198 |  |  |  |
| N (0-15) | 198 | 26.1 | **0.932** | 0.196 |  |  |  |
| N (15-30) | 158 | 24.7 | **0.931** | 0.227 |  |  |  |
| N (30-45) | 149 | 21.4 | **0.927** | 0.279 |  |  |  |
| SMBC | 262 | 37.7 | 0.848 | **0.317** |  |  |  |
| SMBN | 35.9 | 9.29 | 0.911 | **0.360** |  |  |  |
| SMBS | 10.1 | 2.03 | **0.980** | - |  |  |  |
| AS | 114 | 20.0 | **0.905** | - |  |  |  |
| DHA | 10.8 | 1.73 | 0.917 | **0.331** |  |  |  |
| URE | 103 | 23.0 | 0.922 | **0.369** |  |  |  |
| GYM | 4998 | 996 | 0.874 | 0.321 |  |  |  |
| TSU | 12.4 | 4.05 | 0.955 | 0.272 |  |  |  |

**Table 9. Variable scores by linear scoring functions**

| **Variables** | **50% NPK** | **100% NPK** | **150% NPK** | **NPK+HW** | **NPK+ Zn** | **NP** | **N alone** | **NPK+ FYM** | **NPK (-S)** | **Control** |
| --- | --- | --- | --- | --- | --- | --- | --- | --- | --- | --- |
| **PC1** | | | | | | | | | | |
| WSS (15-30) | 0.53 | 0.69 | 0.97 | 0.66 | 0.86 | 0.61 | 0.28 | 1.00 | 0.22 | 0.21 |
| HSS (15-30) | 0.56 | 0.76 | 0.95 | 0.70 | 0.90 | 0.69 | 0.57 | 1.00 | 0.44 | 0.41 |
| OS (0-15) | 0.65 | 0.77 | 0.90 | 0.75 | 0.82 | 0.73 | 0.60 | 1.00 | 0.52 | 0.49 |
| OS (15-30) | 0.61 | 0.76 | 0.94 | 0.74 | 0.79 | 0.71 | 0.52 | 1.00 | 0.45 | 0.44 |
| OS (30-45) | 0.57 | 0.69 | 0.89 | 0.67 | 0.80 | 0.64 | 0.45 | 1.00 | 0.37 | 0.36 |
| TS (0-15) | 0.68 | 0.80 | 0.96 | 0.77 | 0.87 | 0.75 | 0.58 | 1.00 | 0.54 | 0.53 |
| TS (15-30) | 0.55 | 0.69 | 0.85 | 0.68 | 0.76 | 0.62 | 0.47 | 1.00 | 0.40 | 0.39 |
| TS (30-45) | 0.55 | 0.70 | 0.92 | 0.69 | 0.81 | 0.68 | 0.45 | 1.00 | 0.40 | 0.37 |
| SOC (30-45) | 0.82 | 0.89 | 0.99 | 0.89 | 0.93 | 0.86 | 0.79 | 1.00 | 0.87 | 0.72 |
| Soil N (0-15) | 0.70 | 0.78 | 0.96 | 0.83 | 0.88 | 0.80 | 0.77 | 1.00 | 0.81 | 0.65 |
| Soil N (15-30) | 0.70 | 0.80 | 0.86 | 0.73 | 0.77 | 0.69 | 0.64 | 1.00 | 0.75 | 0.58 |
| Soil N (30-45) | 0.71 | 0.86 | 0.98 | 0.84 | 0.88 | 0.78 | 0.68 | 1.00 | 0.86 | 0.65 |
| SMBS | 0.69 | 0.85 | 0.90 | 0.80 | 0.86 | 0.81 | 0.61 | 1.00 | 0.65 | 0.49 |
| AS | 0.67 | 0.78 | 0.72 | 0.76 | 0.79 | 0.76 | 0.61 | 1.00 | 0.62 | 0.54 |
| **PC2** | | | | | | | | | | |
| SOC (0-15) | 0.74 | 0.81 | 0.89 | 0.84 | 0.82 | 0.79 | 0.78 | 1.00 | 0.83 | 0.66 |
| SOC (15-30) | 0.72 | 0.80 | 0.88 | 0.81 | 0.82 | 0.77 | 0.76 | 1.00 | 0.81 | 0.62 |
| SMBC | 0.85 | 0.92 | 0.95 | 0.91 | 0.93 | 0.88 | 0.65 | 1.00 | 0.92 | 0.63 |
| SMBN | 0.57 | 0.81 | 0.94 | 0.80 | 0.87 | 0.71 | 0.54 | 1.00 | 0.83 | 0.38 |
| DHA | 0.64 | 0.80 | 0.83 | 0.76 | 0.79 | 0.76 | 0.69 | 1.00 | 0.78 | 0.54 |
| URE | 0.64 | 0.72 | 0.85 | 0.76 | 0.79 | 0.67 | 0.60 | 1.00 | 0.77 | 0.40 |

**Table 10. Soil Quality indices by Principal Component Analysis in an *Inceptisol***

| **Treatments** | **SQI_W_** | **SQI_A_** |
| --- | --- | --- |
| 50% NPK | 4.44 | 0.22 |
| 100% NPK | 5.34 | 0.27 |
| 150% NPK | 6.30 | 0.31 |
| 100% NPK+HW | 5.19 | 0.26 |
| 100% NPK+ Zn | 5.78 | 0.29 |
| 100% NP | 5.00 | 0.25 |
| 100% N | 3.97 | 0.20 |
| 100% NPK+ FYM | 6.90 | 0.35 |
| 100% NPK (-S) | 3.94 | 0.20 |
| Control | 3.38 | 0.17 |
